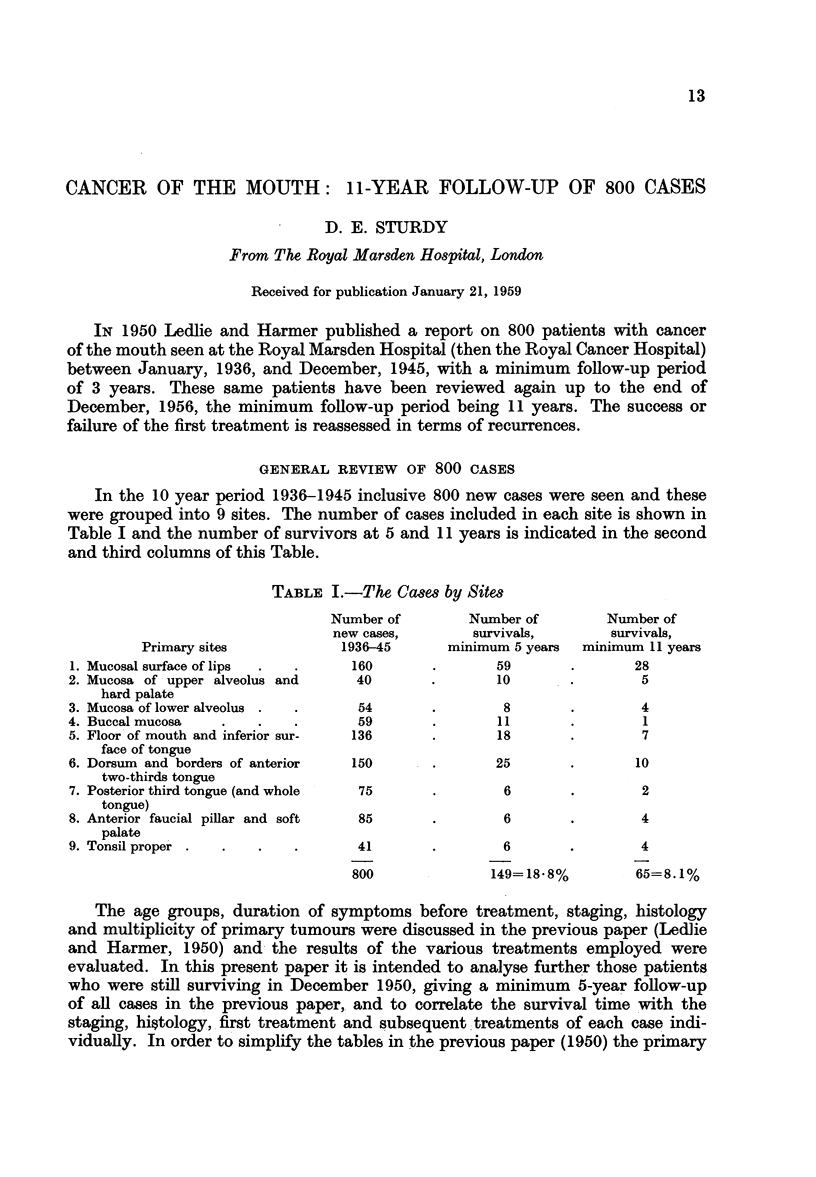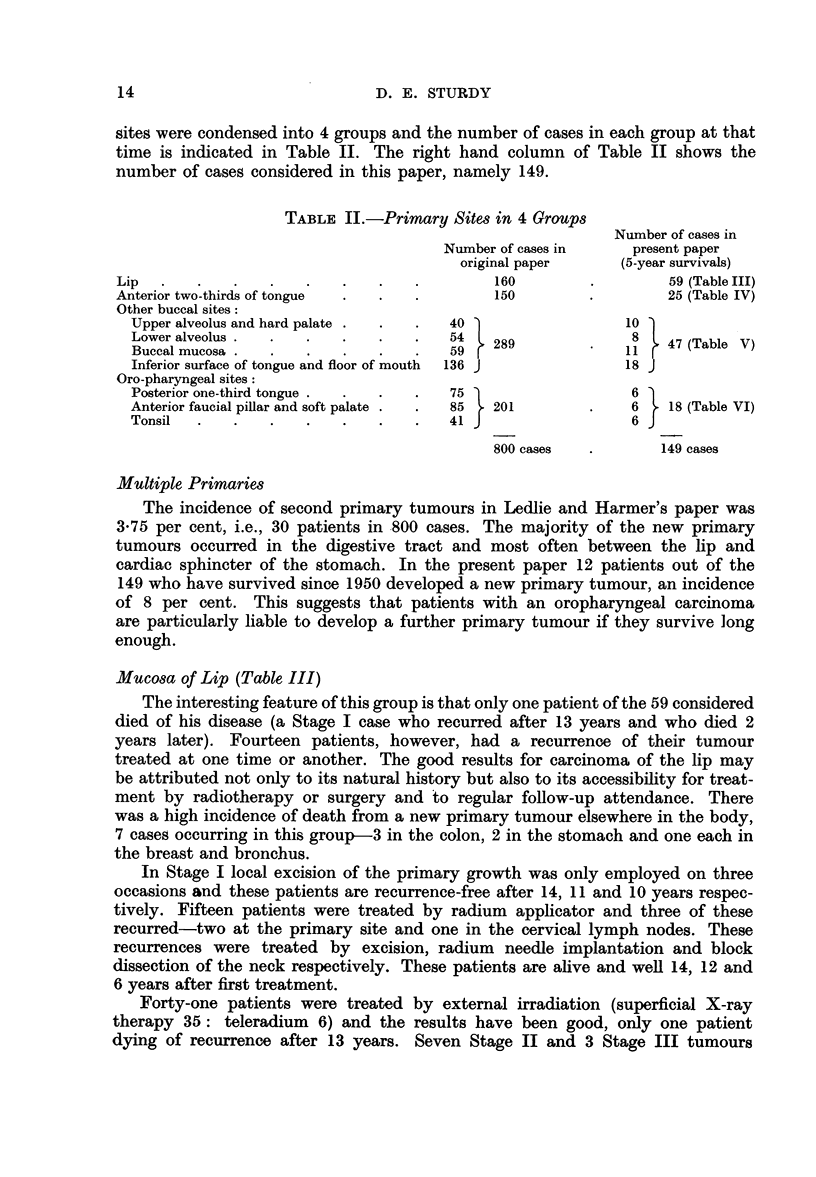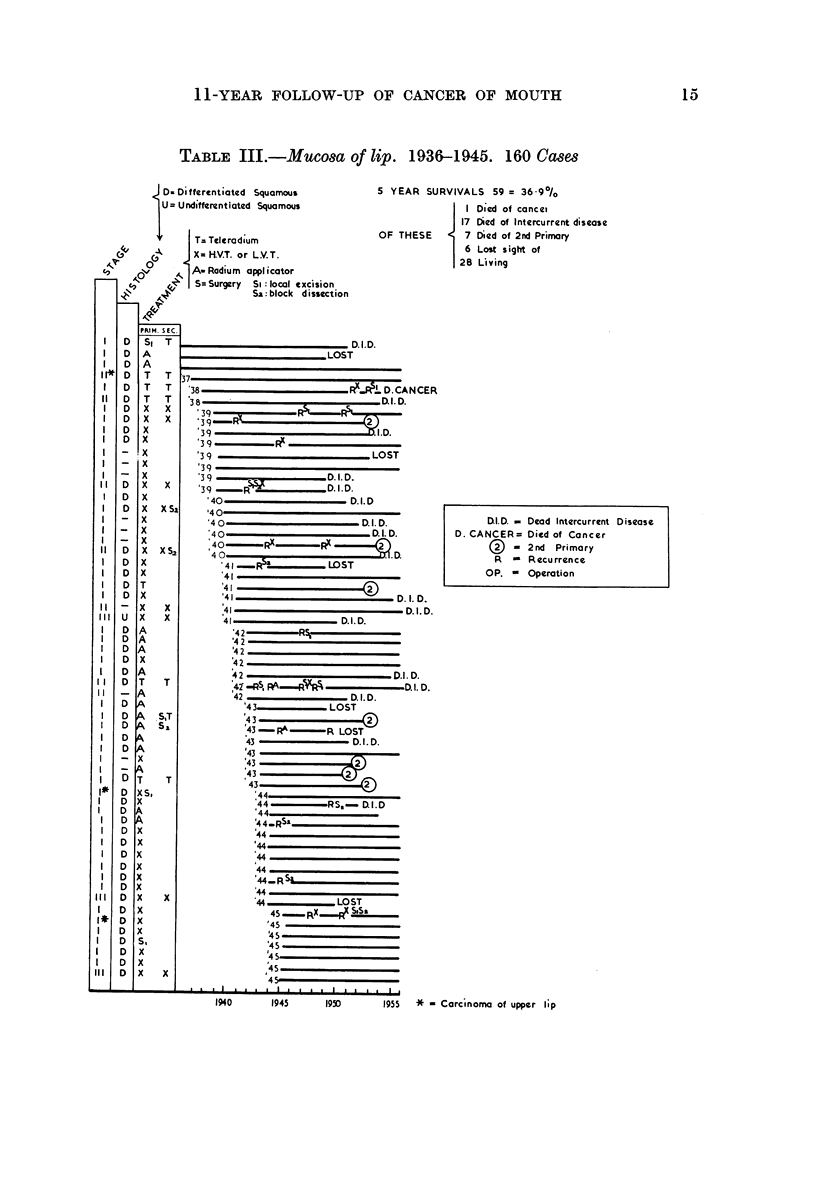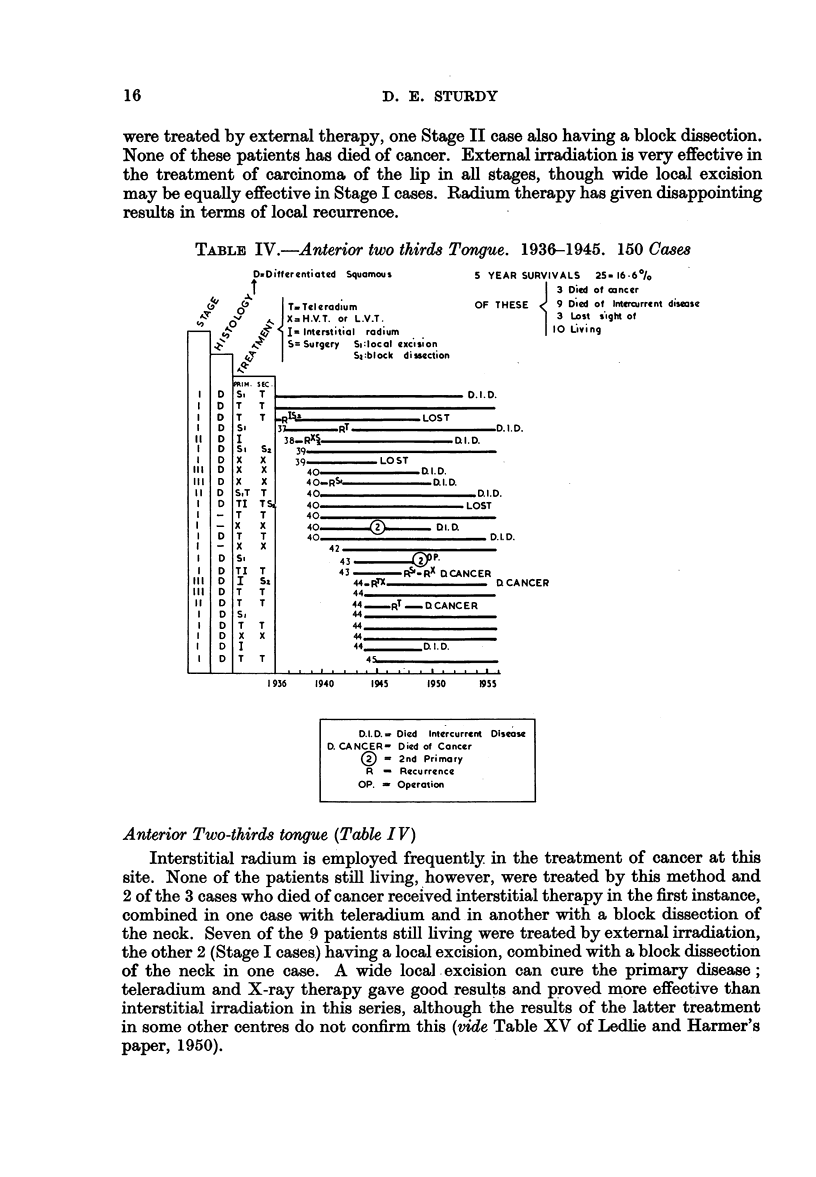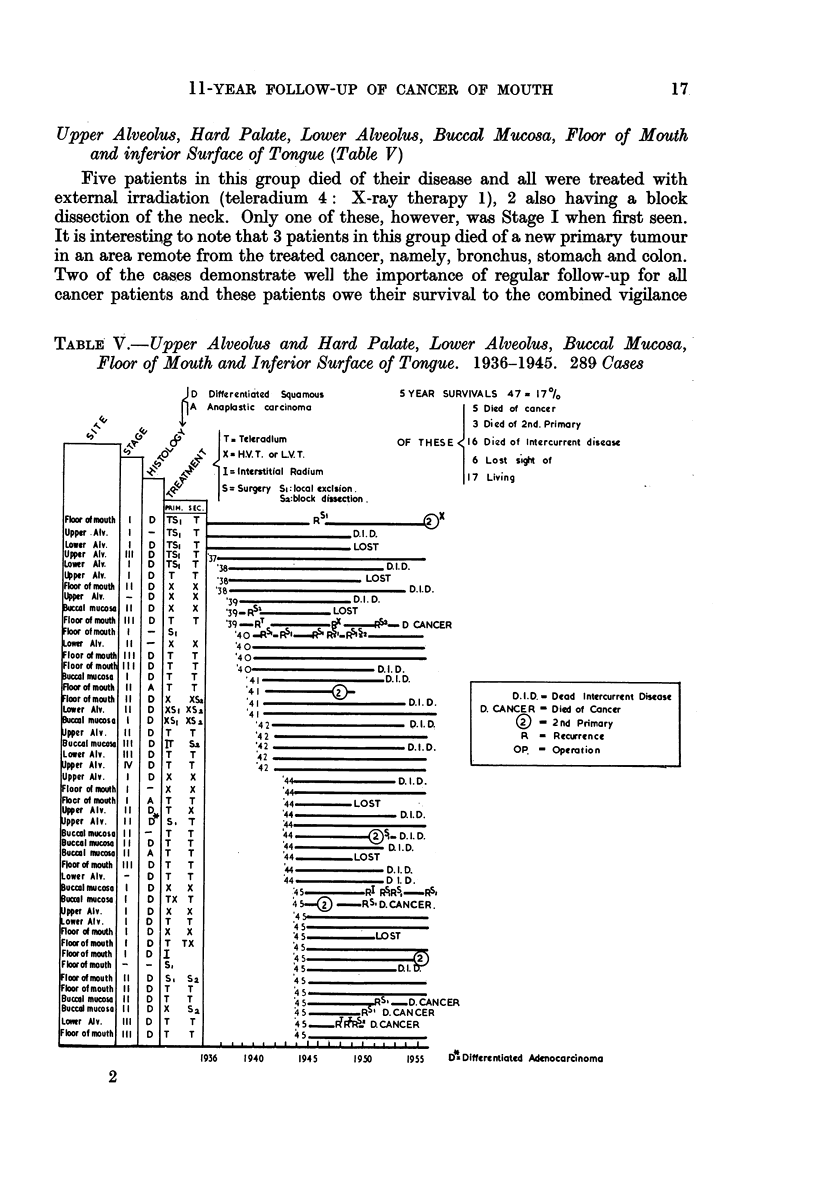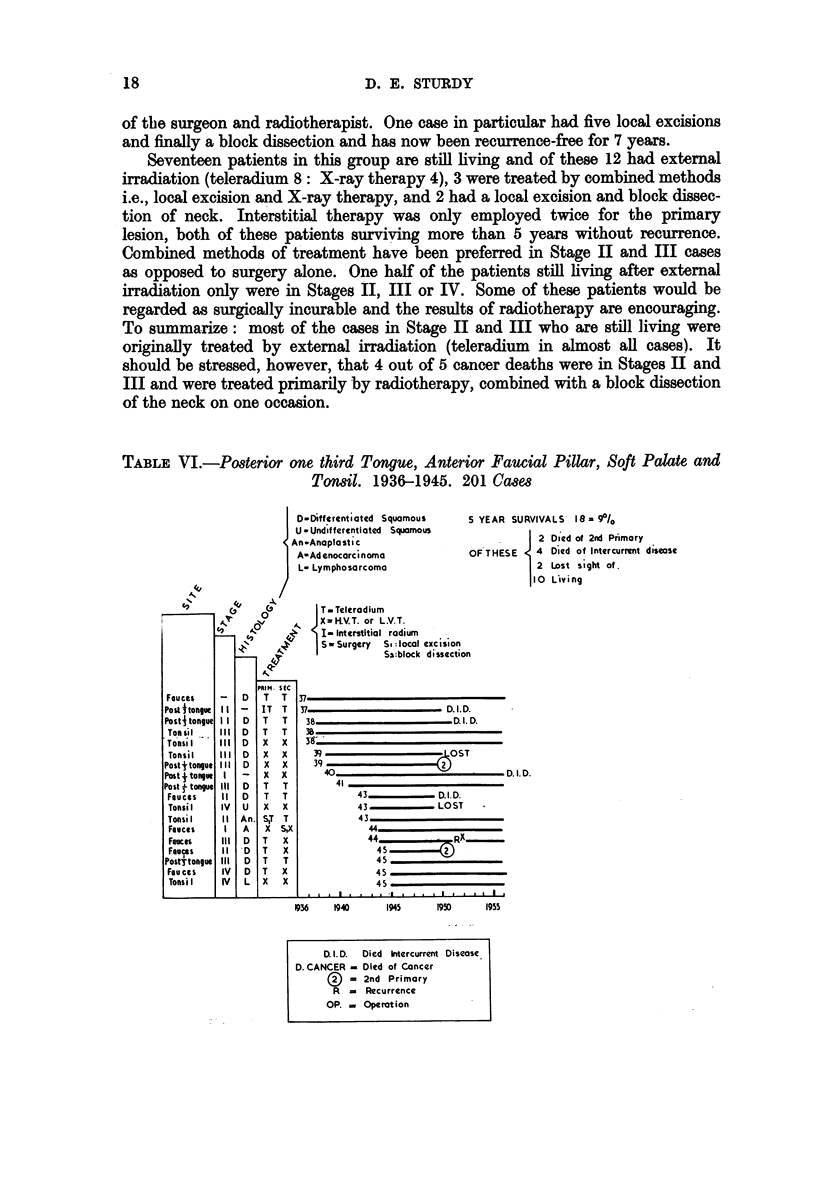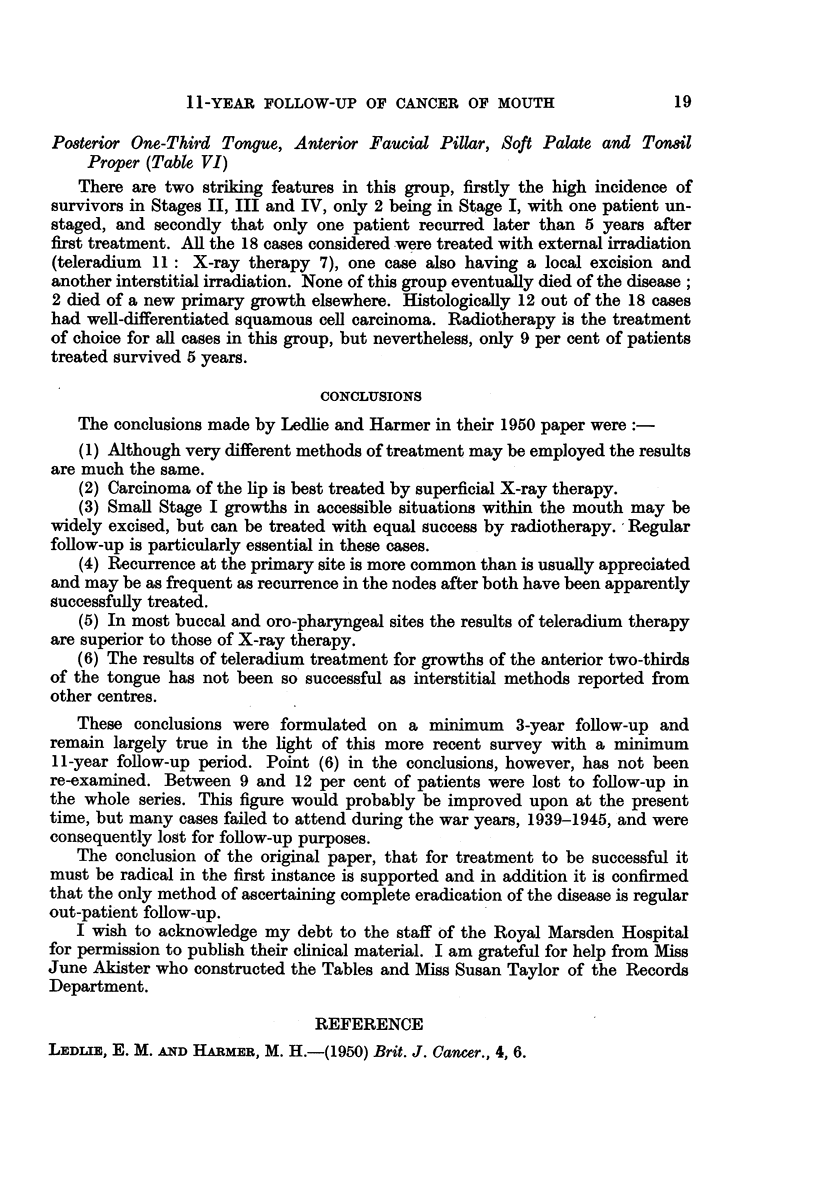# Cancer of the Mouth: 11-Year follow-up of 800 Cases

**DOI:** 10.1038/bjc.1959.2

**Published:** 1959-03

**Authors:** D. E. Sturdy


					
13

CANCER OF THE MOUTH: 11-YEAR FOLLOW-UP OF 800 CASES

D. E. STURDY

From The Royal Marsden Hospital, London

Received for publication January 21, 1959

IN 1950 Ledlie and Harmer published a report on 800 patients with cancer
of the mouth seen at the Royal Marsden Hospital (then the Royal Cancer Hospital)
between January, 1936, and December, 1945, with a minimum follow-up period
of 3 years. These same patients have been reviewed again up to the end of
December, 1956, the minimum follow-up period being 11 years. The success or
failure of the first treatment is reassessed in terms of recurrences.

GENERAL REVIEW OF 800 CASES

In the 10 year period 1936-1945 inclusive 800 new cases were seen and these
were grouped into 9 sites. The number of cases included in each site is shown in
Table I and the number of survivors at 5 and 11 years is indicated in the second
and third columns of this Table.

TABLE I.-The Cases by Sites

Number of        Number of        Number of
new cases,       survivals,        survivals,

Primary sites            1936-45      minimum 5 years  minimum 11 years
1. Mucosal surface of lips  .  .   160       .       59       .       28
2. Mucosa of upper alveolus and     40       .       10       .        5

hard palate

3. Mucosa of lower alveolus .  .    54       .        8       .        4
4. Buccal mucosa   .    .   .       59       .       11       .        1
5. Floor of mouth and inferior sur-  136     .       18       .        7

face of tongue

6. Dorsum and borders of anterior  150       .       25       .       10

two-thirds tongue

7. Posterior third tongue (and whole  75     .        6       .        2

tongue)

8. Anterior faucial pillar and soft  85      .        6       .        4

palate

9. Tonsil proper .  .   .   .       41       .        6       .        4

800              149=18-8%         65=8.1%

The age groups, duration of symptoms before treatment, staging, histology
and multiplicity of primary tumours were discussed in the previous paper (Ledlie
and Harmer, 1950) and- the results of the various treatments employed were
evaluated. In this present paper it is intended to analyse further those patients
who were still surviving in December 1950, giving a minimum 5-year follow-up
of all cases in the previous paper, and to correlate the survival time with the
staging, histology, first treatment and subsequent treatments of each case indi-
vidually. In order to simplify the tables in the previous paper (1950) the primary

D. E. STURDY

sites were condensed into 4 groups and the number of cases in each group at that
time is indicated in Table II. The right hand column of Table II shows the
number of cases considered in this paper, namely 149.

TABLE II.-Primary Sites in 4 Groups

Number of cases in
Number of cases in     present paper

original paper     (5-year survivals)

Lip   .   .   .    .   .   .    .   .         160        .         59 (Table III)
Anterior two-thirds of tongue  .  .  .        150        .         25 (Table IV)
Other buccal sites:

Upper alveolus and hard palate .  .  .  40                  10

Lower alveolus .  .  .    .   .   .    54   289              8   47 (Table V)
Buccal mucosa .  .   ..     .                . 59  1(Table V)11
Inferior surface of tongue and floor of mouth  136 J        18 J
Oro-pharyngeal sites:

Posterior one-third tongue.  .  .  .   75                    6

Anterior faucial pillar and soft palate.  .  85  201    .    6   18 (Table VI)
Tonsil  .   .    .   .    .   .   .    41                    6

800 cases   .       149 cases

Multiple Primaries

The incidence of second primary tumours in Ledlie and Harmer's paper was
3.75 per cent, i.e., 30 patients in 800 cases. The majority of the new primary
tumours occurred in the digestive tract and most often between the lip and
cardiac sphincter of the stomach. In the present paper 12 patients out of the
149 who have survived since 1950 developed a new primary tumour, an incidence
of 8 per cent. This suggests that patients with an oropharyngeal carcinoma
are particularly liable to develop a further primary tumour if they survive long
enough.

Mucosa of Lip (Table III)

The interesting feature of this group is that only one patient of the 59 considered
died of his disease (a Stage I case who recurred after 13 years and who died 2
years later). Fourteen patients, however, had a recurrence of their tumour
treated at one time or another. The good results for carcinoma of the lip may
be attributed not only to its natural history but also to its accessibility for treat-
ment by radiotherapy or surgery and to regular follow-up attendance. There
was a high incidence of death from a new primary tumour elsewhere in the body,
7 cases occurring in this group-3 in the colon, 2 in the stomach and one each in
the breast and bronchus.

In Stage I local excision of the primary growth was only employed on three
occasions and these patients are recurrence-free after 14, 11 and 10 years respec-
tively. Fifteen patients were treated by radium applicator and three of these
recurred-two at the primary site and one in the cervical lymph nodes. These
recurrences were treated by excision, radium needle implantation and block
dissection of the neck respectively. These patients are alive and well 14, 12 and
6 years after first treatment.

Forty-one patients were treated by external irradiation (superficial X-ray
therapy 35: teleradium 6) and the results have been good, only one patient
dying of recurrence after 13 years. Seven Stage II and 3 Stage III tumours

14

15

1 -YEAR FOLLOW-UP OF CANCER OF MOUTH

TABLE III.-Mucosa of lip. 1936-1945. 160 Cases

D= Differentiated Squamous
U= Undifferentiated Squamous

T- Telerad;um

4 &            X = H.V.T. or L.V.T.

AY'     00

',     o?     -\, A- Radium applicator

1 S= Surgery   Si local excision

/ .,~  ...1'       Sa: block dissection

S YEAR SURVIVALS 59 = 36.90/0

I Died of cancei

17 Died of Intercurrent disease
OF THESE        7 Died of 2nd Primary

6 Lost sight of
28 Living

D. .D.
LOST

57v

'38                  .J(5l ?D.CANCER
38             o        DID.

'39   R'

3 9

39                          -.I.D.
39 R

'39                          LOST
'39

'39                  D.I.D.
39          R        D. .D.

'40                     D.I.D
'40

'40                       D.I.D.

'40                        D.I.D.

'40 -        RX-:  O

-(7

40      ,                     D.

'41 IR            LOST
41

'41                    (

'41                          D. l D.

41                             D.I.D.
41-                 D.I.D.

'42        RS,
'4 2
'42
42 2

42                         DI. D.

42 -RS, A--RVNXF             D.. D.
42                  D.I.D.

'4 3          LOST

'43 --
43     RA    R LOST

43                D.I.D.
'43
'43
43

44

44          RS,-- DI.D
44

'4 4-RS'
44
44
44
44

44_RSI
44

44           LOST

45    RX.:   S,S,
'45
'45
'45
'4 5

45-
4E

i I  i i , i I  ,   I  ,,.  .  i

DI.D. - Dead Intercurrent Disease
D. CANCER= Died of Cancer

G    - 2nd Primary
R  -Recurrence
OP. - Operation

1940        1945      1950        1955   * - Carcinoma of upper lip

I

I

PRIM. SEC.

SI T
A
A

T T
T T
T T
x x
X  X
x
x
x
x
x

x x
x

x x s2
x
x
x

x x 52
x
x
T
x

x   x
x   x

A
A
A
x
A
T
A

I,
II

Ill

iii
1I
II
II

I
II

I

II

III

Ill

I*
I
I

Il

D
D
D
D

U
D
D
D
D
D
D
D
D
D
D
D

D
D
D
D
D
D
D
D
D
D
D
D
D
D
D
D
D
D
D

T

S,T
Si,

T

X
X

A
A
A
A
x
A
T

XS,
x

A
A

x
x
x
x

5,

x
x
x

, . I .

. . . .

16                           D. E. STURDY

were treated by external therapy, one Stage II case also having a block dissection.
None of these patients has died of cancer. External irradiation is very effective in
the treatment of carcinoma of the lip in all stages, though wide local excision
may be equally effective in Stage I cases. Radium therapy has given disappointing
results in terms of local recurrence.

TABLE IV.-Anterior two thirds Tongue. 1936-1945. 150 Cases

D Differentiated Squamous        5 y

C         T=Teleradium               OF
"     o   A. X=H.V.T. or L.V.T.

A     ?' In Interstitial radium

I IF       S /= Surgery S,:locol excision

J                SA:block   dissection

PRIM. SEC.

l D So T                                D.Rl.D.
l D T    T

I D T    T           IRS'        LOST
IID S,      37       RT

I  - x   xl   ~ ~ ~ ~~__ _ _ _ _ _ n

lliD I           x                      l38-RX  O . D.

lD S i Si    39--

D X   X    39-          LOST

D X   X      40               D l. D.

D X   X      40-Rs'             Ql. .D.

llD S,T T      40_                      D.I.C

lD TI T S,       40-- LOST

- T T          40                      -

I,v.('"~.4 0 , 2 l~ E1 ".D.

s v   _    _  u *~~~~. v.

I

Ill

I
I

III
III

D
D
D
D
D
D
D
D
D
D
D

T
x

Si
TI

I

T
T
St
T
x

T

T
x

T
SI
T
T

T

x

T

1 936

EAR SURVIVALS      25- 16.6/e

3 Died of cancer

THESE      9 Died of Intercurrent disease

3 Lost sight of
10 Living

D.ID.
D.

D.I.D.- Died Intercurrent Disease
D. CANCER- Died of Cancer

(     = 2nd Primary
R - Recurrence
OP. - Operation

Anterior Two-thirds tongue (Table IV)

Interstitial radium is employed frequently in the treatment of cancer at this
site. None of the patients still living, however, were treated by this method and
2 of the 3 cases who died of cancer received interstitial therapy in the first instance,
combined in one case with teleradium and in another with a block dissection of
the neck. Seven of the 9 patients still living were treated by external irradiation,
the other 2 (Stage I cases) having a local excision, combined with a block dissection
of the neck in one case. A wide local excision can cure the primary disease;
teleradium and X-ray therapy gave good results and proved more effective than
interstitial irradiation in this series, although the results of the latter treatment
in some other centres do not confirm this (vide Table XV of Ledlie and Harmer's
paper, 1950).

40D. D.lD.

42

423         2.P.

43       RI:-RX C CANCER

44- RTX                D CANCER
44

44    RT -   CANCER
44

44
44

44         D.l.D.

4

1940        1945        1950       1955

I            I --                      I        .     .      .     I      .      .       .     .     I     .      .     ,      .      I      .      .     .      .     I      I

11-YEAR FOLLOW-UP OF CANCER OF MOUTH

17..

Upper Alveolus, Hard Palate, Lower Alveolus, Bucca Mucosa, Floor of Mouth

and inferior Surface of Tongue (Table V)

Five patients in this group died of their disease and all were treated with
external irradiation (teleradium 4: X-ray therapy 1), 2 also having a block
dissection of the neck. Only one of these, however, was Stage I when first seen.
It is interesting to note that 3 patients in this group died of a new primary tumour
in an area remote from the treated cancer, namely, bronchus, stomach and colon.
Two of the cases demonstrate well the importance of regular follow-up for all
cancer patients and these patients owe their survival to the combined vigilance

TABLE V.-Upper Alveolus and Hard Palate, Lower Alveolus, Buccal Mucosa,

Floor of Mouth and Inferior Surface of Tongue. 1936-1945. 289 Cases

D   Differentiated  Squamous            S YEAR Sl
A  Anplastic carcinoma

A~       'C,     5

A            6 ..     T Teleradlum                      OF THESE

~.T'  u       O  TES

Co     Ov     _    XaH.V.T. orL.V.T.

I.     <z      I= Interstitlal Radium

A?'~       S=Surgery Sl:locol exclsion.

Sa:block dissection.

Flor of mouth  I  D  TSt T                      R                      X

T
T
T
X
X
T
x
X
x
T

x
T
T

TI

T

XSa
I XSs
I XSI

T

T
T
X
X
T
X
T
T
T
T
T
T
X
T
X
T
X
T

T

Si
T

T
T

I~~~~~~ ~~D.I.D.
~I    -- -   ~LOST
37

38                         D.l.D.
'38                     LOST

'38                             D.I.D.

'39                  D.I.D.
'39-RS-          LOST

'39 -RW           -   -    - D CANCER

'40 _~Rs'_RS-n   -RM2(SS.
'40
'40

'40                    D.. D.

'41                    D.I.D.

4,1

'41-          (~

'41                       D.I.D.
4 1

'42                      D.I.D.
'42

'42                      D.I.D.
42
'42

414                D.I.D.
44.

44         LOST

44                 D.l.D.
44

44                 D. 1. D.
44               D.I.D.
'44  ,      LOST

^44              D.1. D.

44               D l.D.

'4s5        RI RRS, -RS,
4 5-, -    RS' D. CANCER.
'4'
45

'45          LO ST
'45

45
45

4 5         RS    D. CANCER
45         RS' D.CANCER
45-   RTfRS.' D.CANCER
w     wT       Thl         A I I I

,URVIVALS 47 = 17%/

5 Died of cancer

3 Died of 2nd. Primary

E  16 Died of Intercurrent disease

6 Lost sight of
17 Living

D. I.D.- Dead Intercurrent Disease
D. CANCER - Died of Cancer

- 2 nd Primary
R - Recurrence
OP. - Operation

1936   \ 1940        1945        1950       1955     DeDifferentiated Adenocarcinoma

Upper Alv.
Lower AIv.
Upper Alv.
Lower Aiv.
Upper Alv.

Floor of mouth
Upper Aiv.

Icl mucose
loor of mouth
o   of mouth

r Ala.

loor of mouth
loor dof mouth

u l mucosa

or of mouth
or of mouth
Lower Alv.

I mucosa
pper Alv.

Bueol mucosa
Lower AiY.
pper Alv.
Upper Ali.

Floor of mouth
Flocr of mouth

Upper AIv.
pper Ala.

IBuccl mucose

Buccel mcoso
Buccal mucos
Floor of mouth
Lower AIv.

uccol mucosu
Bucal macosu

ppef AIl.
ower Alv.

loor of mouth
Floor of mouth
Floorof mouth
FoIrof mouth

loor of mouth
Floor of mouth
Buccal mucosu
Beccl mucosu
Lower Al .

Floor of mouth

Il
II
III
ll
iii
lill

I1

Ill

II

IIi

Ii
II

III
ll

ll
I I

III
II

I

II
II
I
I I
Ill
I I

I

I

II

I

I,

I

II
II
II
II

III
III

TS
TSi
TSi

X

XS,
X
T

St
x
T
T

T
T
T
T

T
IT
T

X
T

T

fix

T
T
T
T

T
T

X

TX

X
T

X
T
I
so
St
T
T
X
T
T

D
D
D
D
D
D
D
D

D
D
D
A
D
D
D
D
D
D
D
D

A
D

D
A
D
D
D
D
D
D
D
D
D

D
D
D
D
D
D

2

I                                    I

'I

,I

18                           D. E. STURDY

of the surgeon and radiotherapist. One case in particular had five local excisions
and finally a block dissection and has now been recurrence-free for 7 years.

Seventeen patients in this group are still living and of these 12 had external
irradiation (teleradium 8: X-ray therapy 4), 3 were treated by combined methods
i.e., local excision and X-ray therapy, and 2 had a local excision and block dissec-
tion of neck. Interstitial therapy was only employed twice for the primary
lesion, both of these patients surviving more than 5 years without recurrence.
Combined methods of treatment have been preferred in Stage II and III cases
as opposed to surgery alone. One half of the patients still living after external
irradiation only were in Stages II, III or IV. Some of these patients would be
regarded as surgically incurable and the results of radiotherapy are encouraging.
To summarize: most of the cases in Stage II and III who are still living were
originally treated by external irradiation (teleradium in almost all cases). It
should be stressed, however, that 4 out of 5 cancer deaths were in Stages II and
III and were treated primarily by radiotherapy, combined with a block dissection
of the neck on one occasion.

TABLE VI.-Posterior one third Tongue, Anterior Faucial Pillar, Soft Palate and

Tonsil. 1936-1945. 201 Ca8es

-Differentiated Squamous

- Undifferentiated  Squamous
-Anapla sti c

?-Ad enocarci noma
- Lymphosarcoma

5 YEAR SURVIVALS      18= 90/0

2 Died of 2nd Primary

OF THESE      4  Died of Intercurrent disease

2 Lost sight of.
I10 Lsving

T - Teleradium

X= H.VT. or L.V.T.

T.- Interstitial radium

S-Surgery   S local excision

Ss:block dissection

7

7                 DD..D.

3B                D.I.D.
38
38

39-             lOST
39

~~40.~ ~ (O        D. DID.
41

43        D.I.D.
43        LOST

43,

44

44         R
45

45     5
45
45

I    ..   A  .   . . .  .  . .   . .   I

1940           1945          1950         1955

D. 1. D. Died Intercurrent Disease
D. CANCER - Died of Cancer

?    = 2nd Primary
R - Recurrence
OP. - Operation

'C,

Fa uces

Post t tongue
Post~ tongue
Ton si I
Tonsil I
Tonsil I

Post , tongue
Post + tongue
Post ~- tongue
Faouces
Tonsi I
Tonsil I
Fouces
Fauces
Fouc s

PostTtongue
Fou ces
Tonsi I

D

U.
An
A

IL

go      O     .

PRIM  SEC

-   D   T  T  3
i I -  I   T  3 I
1   D   T  T
Ill D   T  T
III D   X  X
III D   X  X
III D   X  X

I  -   X X
III  D  T  T
ll D    T  T
IV  U   X  X
I I An. S,T T

I  A   X SIX
III D  T   X
II  D  T   X
III  D  T  T
IV  D  T   X
IV  L  X   X

1936

- - -

I

11-YEAR FOLLOW-UP OF CANCER OF MOUTH                  19

Posterior One-Thirt Tongue, Anterior Faucial Pillar, Soft Palate and Tonsil

Proper (Table VI)

There are two striking features in this group, firstly the high incidence of
survivors in Stages II, III and IV, only 2 being in Stage I, with one patient un-
staged, and secondly that only one patient recurred later than 5 years after
first treatment. All the 18 cases considered were treated with external irradiation
(teleradium 11: X-ray therapy 7), one case also having a local excision and
another interstitial irradiation. None of this group eventually died of the disease;
2 died of a new primary growth elsewhere. Histologically 12 out of the 18 cases
had well-differentiated squamous cell carcinoma. Radiotherapy is the treatment
of choice for all cases in this group, but nevertheless, only 9 per cent of patients
treated survived 5 years.

CONCLUSIONS

The conclusions made by Ledlie and Harmer in their 1950 paper were:

(1) Although very different methods of treatment may be employed the results
are much the same.

(2) Carcinoma of the lip is best treated by superficial X-ray therapy.

(3) Small Stage I growths in accessible situations within the mouth may be
widely excised, but can be treated with equal success by radiotherapy. Regular
follow-up is particularly essential in these cases.

(4) Recurrence at the primary site is more common than is usually appreciated
and may be as frequent as recurrence in the nodes after both have been apparently
successfully treated.

(5) In most buccal and oro-pharyngeal sites the results of teleradium therapy
are superior to those of X-ray therapy.

(6) The results of teleradium treatment for growths of the anterior two-thirds
of the tongue has not been so successful as interstitial methods reported from
other centres.

These conclusions were formulated on a minimum 3-year follow-up and
remain largely true in the light of this more recent survey with a minimum
11-year follow-up period. Point (6) in the conclusions, however, has not been
re-examined. Between 9 and 12 per cent of patients were lost to follow-up in
the whole series. This figure would probably be improved upon at the present
time, but many cases failed to attend during the war years, 1939-1945, and were
consequently lost for follow-up purposes.

The conclusion of the original paper, that for treatment to be successful it
must be radical in the first instance is supported and in addition it is confirmed
that the only method of ascertaining complete eradication of the disease is regular
out-patient follow-up.

I wish to acknowledge my debt to the staff of the Royal Marsden Hospital
for permission to publish their clinical material. I am grateful for help from Miss
June Akister who constructed the Tables and Miss Susan Taylor of the Records
Department.

REFERENCE

LEDLIE, E. M. AND HARMER, M. H.-(1950) Brit. J. Cancer., 4, 6.